# The effect of source of protein fed during pregnancy and lactation on the development of characteristics of metabolic syndrome in male offspring of obese Wistar rats

**DOI:** 10.1017/jns.2023.97

**Published:** 2023-11-15

**Authors:** Alireza Jahan-mihan

**Affiliations:** Department of Nutrition and Dietetics, Brooks College of Health, University of North Florida, Jacksonville, FL, USA

**Keywords:** Blood pressure, Dietary proteins, Food intake, Gestational obesity, Metabolic syndrome

## Abstract

Gestational obesity has major negative impacts on both mothers and their offspring. More than two-thirds of women of reproductive age in the United States are overweight and/or obese. We previously reported that the source of protein in the maternal diet influences the phenotype of offspring born to normal-weight dams. However, whether it has the same effect in obese mothers was unclear. The casein- and soya protein-based diets were fed to obese pregnant Wistar rats and compared for their effects on characteristics of the metabolic syndrome in male offspring. Dams randomized to either a casein (CD) or soya protein (SD) diet (*n* 12). Pups were weaned to either a CD or SD for 16 weeks. Offspring of SD dams had higher birthweight (*P* < 0⋅01). Glucose metabolism was not altered at birth but fasting blood glucose (FBG) (*P* < 0⋅02), insulin (*P* < 0⋅0002), insulin/glucose ratio (*P* < 0⋅03), and HOMA-IR index (*P* < 0⋅0002) were higher in offspring of SD dams at week 17. The pulse rate was higher in dams (*P* < 0⋅03). Food intake and body weight of offspring were affected by interactive effects of time and dams’ diet (*P* < 0⋅05). Food intake was not influenced by maternal diet, but it was higher in pups weaned to SD dams (*P* < 0⋅03) The results of this study indicate that although the source of protein in the maternal diet is still an influencing factor in the outcome of the pregnancy in obese mothers, gestational obesity may mask this effect possibly by imposing general detrimental effects on measured parameters regardless of the source of protein in maternal diet.

## Introduction

The notion that fetal development can influence adult diseases is a relatively recent concept and it led to a series of studies on the influence of the *in utero* environment on the fetus and neonate that has been grasped in the developmental origin of health and diseases (DOHaD).^(1)^ Developmental plasticity provides an open window for environmental factors (e.g. diet) to permanently alter the development of tissues and organs through an adaptation process to environmental stimuli. The interaction between *in utero* and post-natal environment has been described by the predictive adaptive response (PAR) hypothesis. According to this hypothesis, offspring that received a weaning diet similar to that of their mothers are more likely to adeptly adapt to the post-natal environment compared to those who undergo a different diet from their mothers.^(2)^

More than two-thirds of women of reproductive age (20–39 years old) in the United States are overweight and/or obese, half of whom are obese.^(3)^ Maternal obesity, in parallel, has been turned into one of the most common complications of pregnancy and is a major risk factor for gestational diabetes (GDM).^(3)^ Moreover, maternal obesity or GDM during pregnancy increases the risk of obesity and/or glucose intolerance in offspring.^(4–6)^

Epidemiological evidence indicates an association between increased nutrient supply before birth and later obesity. Intrauterine exposure to maternal obesity is associated with an increased risk of metabolic syndrome^(7)^ and obesity^(8)^ in later life. Obesity in mothers has been associated with gestational hypertension, preeclampsia, GDM, and high fetal birth weights greater than 4000 g. GDM results in hyperglycaemia and hyperinsulinemia in the fetus during late development and a higher risk of obesity in later life compared to infants of nondiabetic mothers.^(5–9)^ Obesity during pregnancy may also influence fetal growth and post-natal outcomes independent of GDM.^(7–10)^ It has been suggested that in obese mothers without clinical signs of GDM, fetal hyperinsulinemia may occur due to maternal mild hyperglycaemia which is below the threshold as defined for GDM.

Beyond their nutritional role as a source of essential amino acids, proteins elicit a wide range of physiological and metabolic functions in a source-dependent manner. They contribute to the regulation of food intake, body weight (BW), glucose and lipid metabolism, and blood pressure. Proteins exhibit their functions in a source-dependent manner. Characteristics of proteins including amino acid composition,^(11–17)^ amino acid sequence, and bioactive peptides (BAPs)^(18–30)^ encrypted in protein structures and their digestion kinetics^(31–35)^ are determinant factors in their effects.

We previously reported that casein- and soya protein-based diets fed during pregnancy and lactation are different in their effect on the development of characteristics of metabolic syndrome in rat dams and their offspring.^(36–38)^ Maternal diet had no effect on dams’ BW but altered their fasting plasma glucose during pregnancy and also altered plasma insulin concentration at week 6 after weaning. They were higher in dams fed the soya protein diet.^(36–38)^ Moreover, food intake, BW, body composition, glucose metabolism, and blood pressure were altered later in life. At the end of the study, offspring born to the dams fed the soya diet had higher BW, body fat, blood pressure, and homeostatic model of assessment of insulin resistance (HOMA-IR) index. The results of these experiments support the hypothesis that nutritionally complete diets differing in protein sources and fed during gestation and lactation differ in their effects on characteristics of the metabolic syndrome in offspring. However, to the best of our knowledge, no study examined the effect of the source of protein consumed during pregnancy in obese pregnant mothers on the health of mothers and children. Therefore, this study, for the first time, examined the effect of both source and quantity of protein fed during pregnancy on mothers’ health and their offspring.

The primary objective of this study was to examine the developmental origin of health and diseases (DOHaD) hypothesis and more specifically whether nutritionally balanced diets that are different in protein sources and fed during gestation and lactation have different effects on mothers’ health and on the risk of development of characteristics of the metabolic syndrome in the offspring. Therefore, obese Wistar rat dams were fed either the AIN-93G soya protein (SD) or casein (CD) diet. Additionally, according to the PAR Hypothesis, the effect of the maternal diet on the offspring is weakened or eliminated if the diet of the offspring is matched with the dams’ diet, two groups of offspring from both maternal groups were fed either a soya protein (SD) or casein (CD) diet. Therefore, the secondary objective of this study was to test the effect of the protein source of the offspring's diet on the consequences of the dams’ diets on the offspring.

We used Wistar rats as a model for this study. Wistar rats have been used in numerous studies examining metabolic syndrome and food intake regulation due to the similarity in metabolic and physiologic mechanisms to humans. Moreover, a well-controlled environment helps to minimize the effect of outliers. In this study, we utilized only male offspring to be consistent and comparable with previous studies conducted in our lab.

## Materials and methods

### Ethical statement

The experiment with Wistar rats followed the National Institutes of Health guide for the care and use of laboratory animals (NIH Publications No. 8023, revised 1978). All experimental procedures involving animals were approved by the University of North Florida Institutional Animal Care and Use Committee (IACUC) (Protocol No: IACUC#16-001).

### Experimental design

A power analysis was performed based on data from a previous study^(37)^ and based on statistical power (80 %) and the two-sided significance level (0⋅05). Newly obese pregnant Wistar rats (*n* 24) were allocated to two groups (*n* 12 per group) and received either a casein-based diet (CD) or a soya protein-based diet (SD) during pregnancy and lactation. The BW of the dams and their offspring was measured weekly. Moreover, the BW of the pups was measured at birth (day 1, after litters were culled to ten pups per dam). To examine the PAR hypothesis, at weaning, one male offspring from each dam on each maternal diet group was allocated to either the CD or SD (*n* 12 per group). BW was measured weekly for 17 weeks after weaning. Systolic blood pressure (SBP) and diastolic blood pressure (DBP), pulse rate, fasting blood glucose (FBG), and blood glucose (BG) response to a glucose load as known as oral glucose tolerance test (OGTT) were measured at weeks 4, 8, 12, and 16 PW. They were also measured at week 6 PW for the dams. Fat pad mass was measured at week 6 PW for the dams and at week 17 PW for offspring (at the termination point). Plasma glucose and insulin were measured at birth, weaning, and at week 17 PW for the offspring and at week 6 PW for the dams.

### Animals and diets

First-time obese pregnant Wistar rats were received at day 3 of gestation (Charles River, NC, USA). They were housed individually in ventilated plastic cages (with bedding) at 22 ± 1 °C and 12-h light–dark cycle (lights off at 09.00 to 21.00 h). The diets (in glass jars) and water were provided *ad libitum*. The well-being of rats was assessed through physical assessments that were carried out prior to, during, or after procedures. No substantial adverse events were notified in any group throughout the study.

Standard diets (AIN-93G) were supplied by Dyets (Dyets Inc. Bethlehem, Pa, USA). The diets and amino acid composition (per kilogram diet) of the diets are shown in Tables 1 and 2, respectively. The protocol was approved by the University of North Florida Institutional Animal Care and Use Committee.

**Table 1.** Composition of the casein and soy protein diets

	Casein diet	Soy protein diet
Cal, g/kg	3597	3780
Casein	200	0
Soy protein	0	200
Sucrose	100	90
Soybean oil	70	70
t-Butyhydroquinone	0⋅014	0⋅005
Cornstarch	397	395
Dyetrose	132	132
Cellulose	50	50
Mineral mix	35	35
Vitamin mix	10	10
Choline bitartrate	2⋅5	2⋅5
L-Cystine	3	2⋅5

**Table 2.** Amino acid composition of casein and soya protein AIN-93G diets

	Micellar casein	Isolated soya protein
Arginine	5⋅7	11⋅9
Aspartic acid	10⋅6	18
Cystine	3⋅5	4⋅5
Glutamic acid	34⋅8	29⋅9
Glycine	2⋅8	6⋅7
Histidine	4⋅4	4⋅1
Isoleucine	8⋅7	7⋅7
Leucine	13⋅9	12⋅8
Lysine	12⋅2	9⋅9
Methionine	4	4⋅5
Phenylalanine	7⋅7	8⋅1
Proline	16	7⋅9
Serine	8⋅4	8⋅1
Threonine	6⋅6	5⋅9
Tryptophan	1⋅7	2⋅2
Tyrosine	8	5⋅9
Valine	10⋅4	7⋅9

Values (g/kg diet) are totals of the addition plus the bound amino acids. Amino acid content of the diets is calculated on the basis of the purity of the protein sources (87 and 90 % for casein and soya protein, respectively). Diets were obtained from Dyets.

### Procedures

#### Food intake

Food intake was measured weekly by weighing the food containers at the beginning and at the end of each week for 16 weeks. Spillage was measured and deducted to calculate the actual food intake.

#### Glucose tolerance test

Rats were fasted overnight for 12 h. Blood samples were taken from the tail vein at fasting and at 15, 30, and 60 min after a glucose administration (0⋅375 g glucose per ml, 5 g glucose per kg BW).^(36)^

#### Blood pressure

SBP, DBP, and pulse were measured by a non-invasive tail-cuff method (optical plethysmography) throughout a tail manometer tachometer system (BP-2000, Visitech system; Apex, NC, USA) at week 6 PW in dams and at weeks 4, 8, 12, and 16 PW in offspring. Rats were restrained in holders adjusted based on their size on a suitably warm platform (30 °C). There was an adaptation process for 5 d. After adaptation to the procedure, on the day of measurement, five mock measurements preceded a series of ten measurements that were used to calculate the average as reported previously.^(36)^

#### Blood collection

Trunk blood was collected in chilled vacutainer tubes (BD, Franklin Lakes, NJ, USA) containing EDTA + Trasylol ® (Bayer AG, Leverkusen, Germany) solution (10 % blood volume, 5 × 108 IU L). Afterward, blood samples were centrifuged at 3000 g and 4 °C for 10 min. Plasma was separated and immediately stored at 70 °C.^(36)^

#### Blood glucose

Blood was taken from the tail vein and glucose concentration was assayed using a hand-held commercial glucometer (Contour ® Next Blood Glucose Meter, Bayer Healthcare LLC, Mishawaka, IN, USA) using test strips. Control solutions (levels 1 and 2) provided by the manufacturer (Bayer, Bayer Healthcare LLC, Mishawaka, IN, USA) were used to test the accuracy and variance of the glucometer and test strips.^(36)^

#### Hormone assays

Plasma insulin concentrations were measured using enzyme-linked immunosorbent assay (catalog no. 80- INSRT-E01, Alpco Diagnostics, Salem, NH, USA) with an assay sensitivity of 0⋅124 ng ml.

#### Body composition

Body composition was assessed by measuring fat mass and lean mass right after killing at weeks 6 for dams and 17 PW for pups. Fat mass was measured by dissection of extracted abdominal, epididymal, and perirenal fat.^(36)^

#### Statistical analyses

The main and interactive effects of the maternal and weaning diets on BW, glucose response, SBP, and DBP were analysed by two-way analysis of variance (ANOVA). Repeated measures were made over time on BW, food intake, SBP, DBP, pulse, BG response, and FBG. The PROC MIXED procedure was used with maternal diets, weaning diets, and time as the main factors. When interactions were statistically significant, a one-way ANOVA followed by *post hoc* Tukey's test was conducted to evaluate treatment effects. The effects of the maternal diets on plasma measures were compared by using a student's unpaired *t*-test. BG response was calculated as the total area under the curve (tAUC) of the BG concentration over 1 h after receiving glucose administered for the glucose tolerance test. The homeostasis model assessment of insulin resistance (HOMA-IR) index was calculated as fasting glucose multiplied by fasting insulin divided by 22⋅5. Data are stated as means with standard errors. Statistical significance was defined at *P* < 0⋅05. All analyses were conducted using SAS (version 9.4; SAS Institute, Cary, NC, USA).

## Results

### Dams

**Fig. 1.**
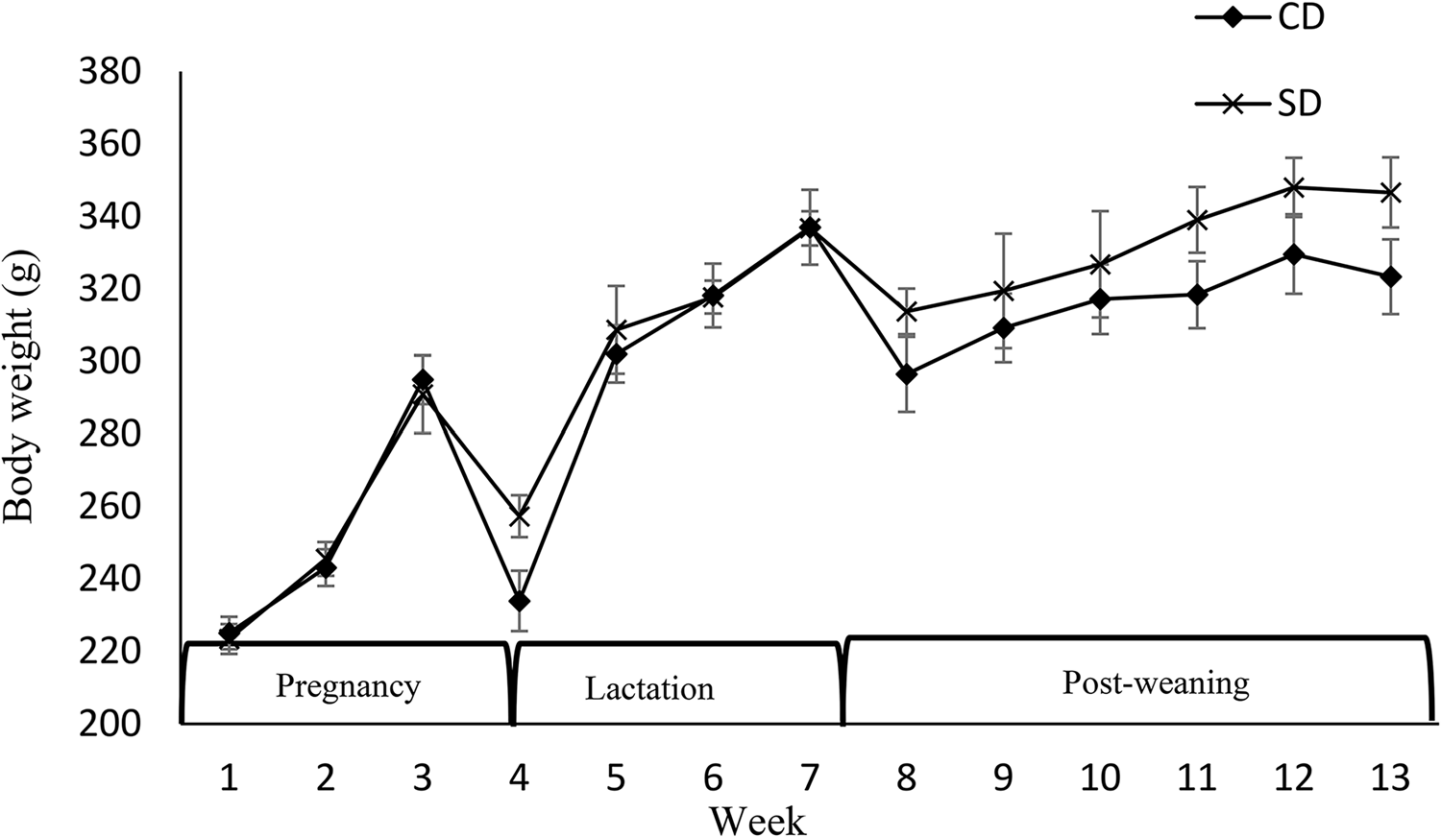
Effect of protein source during gestation on dams’ body weight (BW). CD, casein diet; SD, soya protein diet. Values are means, with their standard errors represented by vertical bars (*n* 12). BW was analysed by MIXED model followed by Tukey's *post hoc* test with diet and time as main factors: diet (NS); time (*P*, 0⋅0001).

**Fig. 2.**
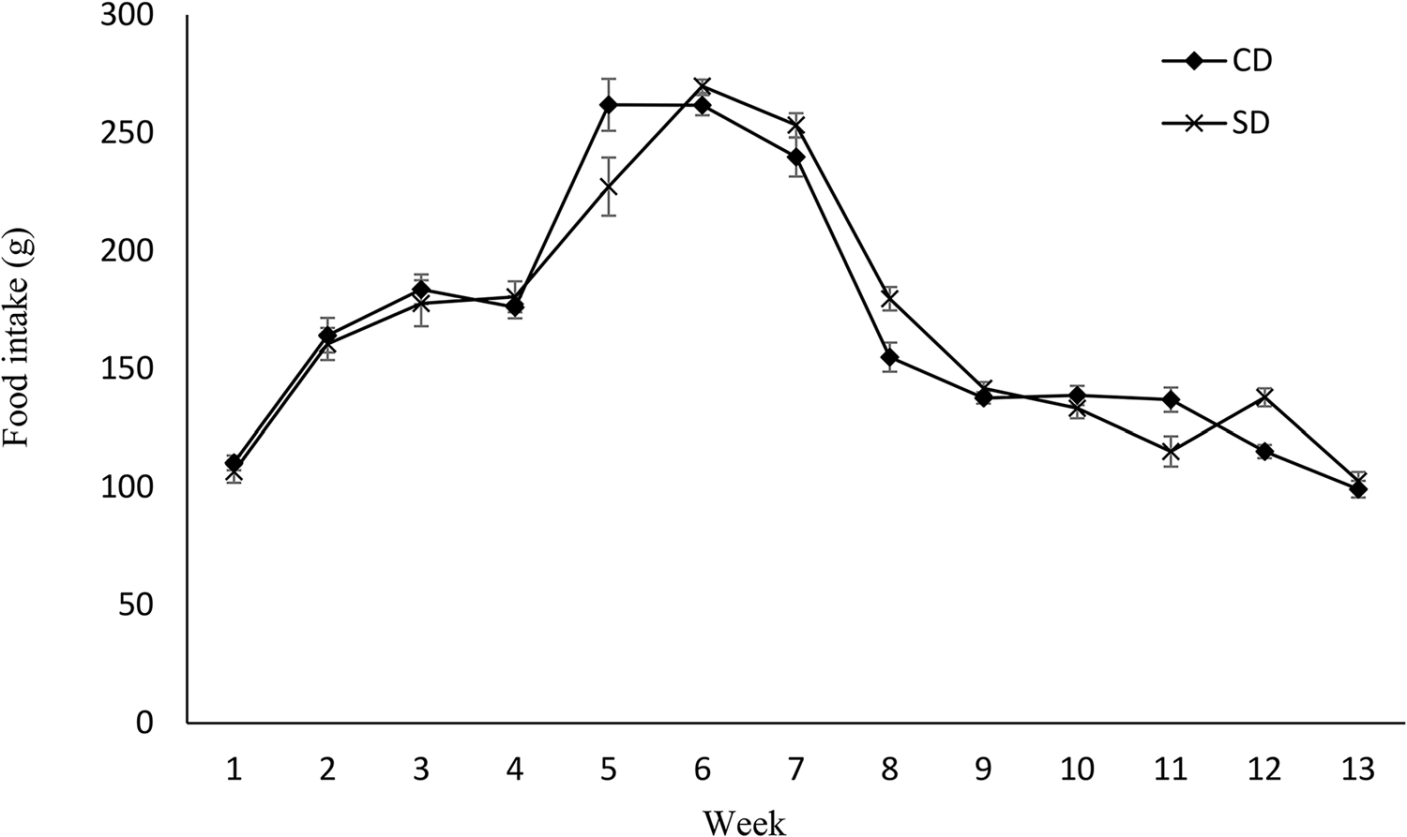
Effect of protein source during gestation on dams’ food intake. CD, casein diet; SD, soya protein diet. Values are means, with their standard errors represented by vertical bars (*n* 12). Food intake was analysed by MIXED model followed by Tukey's *post hoc* test with diet and time as main factors: diet (NS); time (*P*, 0⋅0001).

Dams’ BW was not affected by the diet during pregnancy, after parturition, during lactation, and during PW (Fig. 1). Similarly, no effect of maternal diet on food intake was observed except for week 2 PW when food intake was higher in mothers fed a soya protein-based diet compared with mothers fed a CD (*P* < 0⋅05) (Fig. 2). No difference in fat, and fat/weight percentage at week 7 PW was observed (Table 3). No effect of the diet on SBP and DBP in mothers was observed (data not shown), but the pulse rate was higher in the CD group compared with the SD group (494⋅46 ± 11⋅97 *v.* 458⋅41 ± 9⋅42, respectively) (*P* < 0⋅03). No effect of the maternal diet on mothers’ FBG, BG response, HOMA-IR index, or insulin/glucose ratio at week 7 PW was observed (Table 4).

**Table 3.** Body weight, fat, and fat/weight ratio of dams (week 11) and pups at birth, at weaning and at week 17 PW

Maternal diet	CD	SD	*P*
Dams (Week 11 postpartum)	(g)		
Body weight	326⋅62 ± 16⋅28	347⋅29 ± 7⋅67	NS
Fat	19⋅70 ± 4⋅57	22⋅81 ± 4⋅58	NS
Fat/weight ratio (%)	5⋅82 ± 1⋅02	6⋅74 ± 1⋅30	NS
Pups (at Birth)			
Body weight	6⋅03 ± 0⋅12	6⋅82 ± 0⋅27	*P* < 0⋅05
Weaning			
Body weight	52⋅78 ± 1⋅85	59⋅61 ± 2⋅20	*P* < 0⋅03
Fat	3⋅21 ± 0⋅29	4⋅32 ± 0⋅26	*P* < 0⋅008
Fat/weight ratio (%)	6⋅00 ± 0⋅39	7⋅20 ± 0⋅24	*P* < 0⋅02
Pups (Week 17 PW)			
Body weight	592⋅92 ± 21⋅86	609⋅45 ± 21⋅86	NS
Fat	23⋅21 ± 0⋅95	24⋅50 ± 0⋅77	NS
Fat/weight ratio (%)	3⋅94 ± 0⋅09	4⋅07 ± 0⋅13	NS

CD, casein diet; SD, soya protein diet; NS, not significant; PW, post-weaning.

**Table 4.** Effect of protein source in diets of dams and offspring on fasting plasma measures in the offspring^a^ (mean values with their standard errors, *n* 5–6)

Maternal diet	CD	SD	*P*
Dams (Week 11 postpartum)			
Glucose, mM	5⋅34 ± 0⋅21	5⋅95 ± 0⋅33	NS
Insulin, ng/ml	2⋅46 ± 0⋅21	2⋅74 ± 0⋅17	NS
I/G ratio	0⋅46 ± 0⋅24	0⋅47 ± 0⋅28	NS
HOMA-IR^a^	0⋅59 ± 0⋅71	0⋅74 ± 0⋅72	NS
OGTT *(Week 4 pp)*	445⋅28 ± 14⋅15	476⋅94 ± 15⋅59	NS
Pups			
At birth			
Glucose, mM	5⋅16 ± 0⋅67	5⋅26 ± 0⋅28	NS
Insulin, ng/ml	1⋅34 ± 0⋅26	1⋅28 ± 0⋅29	NS
I/G ratio	0⋅25 ± 0⋅13	0⋅24 ± 0⋅11	NS
HOMA-IR	0⋅28 ± 0⋅06	0⋅27 ± 0⋅05	NS
At weaning			
Glucose, mM	5⋅98 ± 0⋅29	5⋅06 ± 0⋅27	*P* < 0⋅03
Insulin, ng/ml	2⋅71 ± 0⋅13	2⋅73 ± 0⋅12	NS
I/G ratio	0⋅47 ± 0⋅03	0⋅56 ± 0⋅03	*P =* 0⋅05
HOMA-IR	0⋅72 ± 0⋅05	0⋅61 ± 0⋅04	NS
At week 17 PW			
Glucose, mM	4⋅46 ± 0⋅13	4⋅93 ± 0⋅14	*P* < 0⋅02
Insulin, ng/ml	2⋅61 ± 0⋅12	3⋅25 ± 0⋅09	*P* < 0⋅0002
I/G ratio	0⋅59 ± 0⋅03	0⋅67 ± 0⋅02	*P* < 0⋅03
HOMA-IR	0⋅52 ± 0⋅03	0⋅72 ± 0⋅03	*P* < 0⋅0002

CD, casein diet; SD, soya protein diet; HOMA-IR, homeostasis model assessment of insulin resistance; PW, post-weaning

a HOMA-IR index was calculated as fasting glucose (mM) multiplied by fasting insulin (ng/ml) divided by 22⋅5.

### Pups

**Fig. 3.**
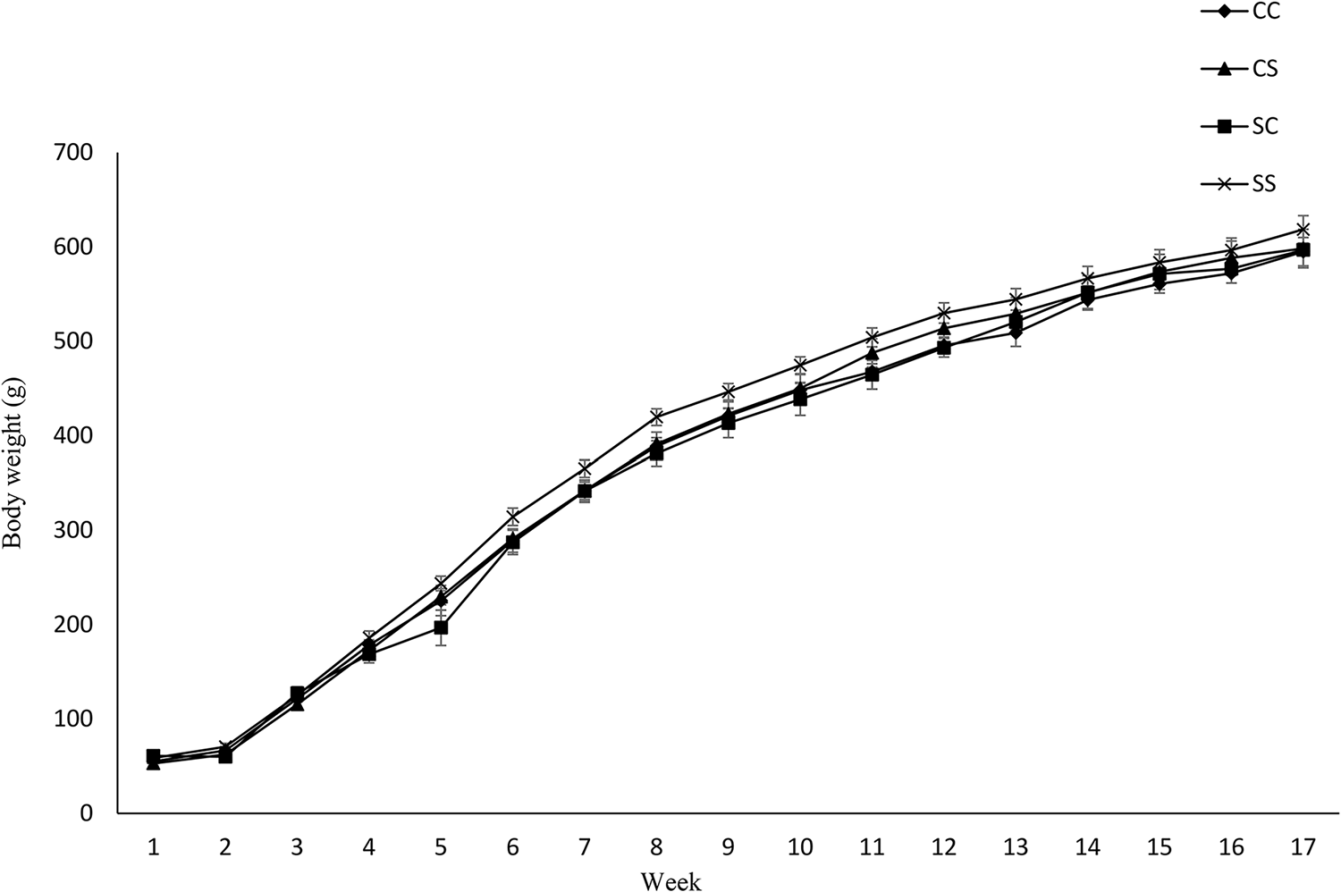
Effect of protein source during gestation on post-weaning body weight (BW) of male offspring. CC, maternal and weaning casein diet; CS, maternal casein and weaning soya protein diet; SC, maternal soya protein and weaning casein diet; SS, maternal and weaning soya protein diet; M, maternal diet; W, weaning diet; NS, not significant values are means, with their standard errors represented by vertical bars (*n* 12). BW was analysed by MIXED model followed by Tukey's *post hoc* test with gestational diet, weaning diet and time as main factors: gestational diet (NS); weaning diet (NS); time (*P*, 0⋅0001); gestational diet × time (*P*, 0⋅05).

**Fig. 4.**
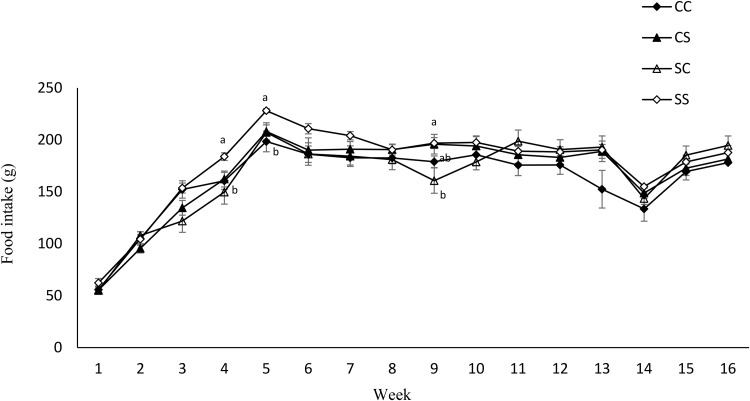
Effect of protein source during gestation on post-weaning food intake of male offspring. CC, maternal and weaning casein diet; CS, maternal casein and weaning soya protein diet; SC, maternal soya protein and weaning casein diet; SS, maternal and weaning soya protein diet; M, maternal diet; W, weaning diet; NS, not significant values are means, with their standard errors represented by vertical bars (*n* 12). Food intake was analysed by MIXED model followed by Tukey's *post hoc* test with gestational diet, weaning diet and time as main factors: gestational diet (NS); weaning diet (*P*, 0⋅03); time (*P*, 0⋅0001); gestational diet × time (*P*, 0⋅001).

Maternal diet altered the birth weight. It was higher in pups born to dams fed SD (6⋅03 g ± 0⋅12 *v.* 6⋅82 g ± 0⋅27) (*P* = 0⋅01) (Table 4). However, no effect of maternal diet on indicators of glucose metabolism was observed at birth (data are not shown). At weaning, BW and fat/weight ratio were influenced by maternal diet, and they were higher in pups born to SD dams (*P* < 0⋅03 and *P* < 0⋅02, respectively). No effect of either maternal or weaning diet on the BW of pups after weaning was observed (Fig. 3). Food intake was not influenced by maternal diet, but it was higher in pups weaned to SD dams (*P* < 0⋅03) (Fig. 4). Moreover, there was a significant interaction between maternal and weaning diets on food intake (*P* = 0⋅002). Food intake was higher in pups weaned to SD dams at weeks 4, 5, and 9 PW.

Glucose metabolism was not altered at birth (Table 4). However, at weaning, FBG was higher in pups born to CD dams (*P* < 0⋅03) while insulin/glucose ratio was relatively higher in pups born to SD dams (*P* = 0⋅05) (Table 4). Fasting plasma glucose (*P* < 0⋅02), insulin (*P* < 0⋅0002), insulin/glucose ratio (*P* < 0⋅03), and HOMA-IR index (*P* < 0⋅0001) were higher in offspring born to S diet-fed dams at week 17 PW (Table 5). There was no effect of either maternal or weaning diet on glucose response to a glucose load, but the interactive effect of maternal and weaning diets was significant (*P* < 0⋅03) (Table 6).

**Table 5.** Effect of protein source in diets of dams and offspring on fasting blood glucose, insulin, insulin/glucose ratio and HOMA-IR index at week 17 (Mean values with their standard errors, *n* 8–12)

Maternal diet	CD		SD		*P*
Weaning diet	CD	SD	CD	SD	
Glucose, mM	4⋅70 ± 0⋅16	4⋅23 ± 0⋅19	4⋅75 ± 0⋅26	5⋅11 ± 0⋅13	M: *P* < 0⋅02; M × W: *P* < 0⋅03
Insulin, ng/ml	2⋅63 ± 0⋅18	2⋅59 ± 0⋅16	3⋅21 ± 0⋅16	3⋅29 ± 0⋅11	M: *P* < 0⋅0002
I/G ratio	0⋅56 ± 0⋅03	0⋅62 ± 0⋅44	0⋅68 ± 0⋅24	0⋅65 ± 0⋅03	M: *P* < 0⋅03
HOMA-IR^a^	0⋅56 ± 0⋅05	0⋅49 ± 0⋅40	0⋅69 ± 0⋅06	0⋅75 ± 0⋅03	M: *P* < 0⋅0002

CD, casein diet; SD, soya protein diet; HOMA-IR, homeostasis model assessment of insulin resistance. MIXED model with dams and pups diets as main factors.

a HOMA-IR index was calculated as fasting glucose (mM) multiplied by fasting insulin (ng/ml) divided by 22⋅5.

**Table 6.** Effect of protein source in diets of dams and offspring on fasting blood glucose and blood glucose response during the oral glucose tolerance tests^a^ (mean values with their standard errors, *n* 10–12)

		CD		SD		*P*
Maternal diet						
Weaning diet		CD	SD	CD	SD	
	Week					
FBG	4	6⋅49 ± 0⋅44	5⋅86 ± 0⋅11	6⋅58 ± 0⋅14	5⋅89 ± 0⋅09	M: *P =* 0⋅06
	8	5⋅55 ± 0⋅53	6⋅12 ± 0⋅61	5⋅55 ± 0⋅44	5⋅59 ± 0⋅45	W: NS
	12	5⋅73 ± 0⋅27	4⋅98 ± 0⋅13	5⋅86 ± 0⋅56	5⋅75 ± 0⋅28	T: *P <* 0⋅03
	16	5⋅49 ± 0⋅21	5⋅74 ± 0⋅17	6⋅69 ± 0⋅37	6⋅91 ± 0⋅23	M × T: *P <* 0⋅04
OGTT	4	644⋅86 ± 43⋅56	526⋅72 ± 22⋅20	576⋅87 ± 25⋅12	708⋅67 ± 59⋅93	M: NS
	8	593⋅88 ± 47⋅70	594⋅70 ± 28⋅57	545⋅76 ± 19⋅32	654⋅99 ± 55⋅84	W: NS
	12	551⋅86 ± 75⋅17	490⋅55 ± 15⋅61	587⋅13 ± 23⋅64	505⋅82 ± 24⋅15	M × W: *P* < 0⋅03
	16	681⋅81 ± 38⋅16	669⋅49 ± 66⋅95	596⋅67 ± 54⋅64	739⋅49 ± 52⋅04	

CD, casein diet; SD, soya protein diet; OGTT, oral glucose tolerance test; FBG, fasting blood glucose; T, time

a MIXED model with D and W as main factors.

GTT: after overnight fasting, rats received glucose (0⋅375 g glucose/ml, 5 g glucose/kg body weight) by oral administration and blood glucose was measured before and 15, 30 and 60 min later.

There was no maternal or weaning diet effect on SBP or DBP. However, maternal and weaning diets had an interactive effect on SBP (*P* < 0⋅03). Pulse was also relatively higher in pups born to dams fed a CD (*P* = 0⋅07) (Table 7).

**Table 7.** Effect of protein source in diets of dams and offspring on systolic blood pressure (SBP) and diastolic blood pressure (DBP) and pulse rate^a^ (mean values with their standard errors, *n* 11–12)

Maternal diet		CD		SD		*P*
Weaning diet		CD	SD	CD	SD	
	Week					
SBP, mmHg	4	116⋅23 ± 7⋅31	135⋅81 ± 6⋅50	123⋅54 ± 3⋅31	124⋅79 ± 5⋅31	M: NS
	8	141⋅34 ± 4⋅02	146⋅37 ± 4⋅82	140⋅71 ± 3⋅34	129⋅87 ± 3⋅38	W: NS
	12	156⋅48 ± 3⋅04	150⋅41 ± 3⋅77	145⋅49 ± 4⋅50	153⋅32 ± 3⋅71	M × W: *P* < 0⋅03
	16	139⋅34 ± 5⋅61	145⋅87 ± 1⋅86	146⋅85 ± 3⋅88	140⋅46 ± 3⋅14	T: *P* < 0⋅0001
DBP, mmHg	4	77⋅82 ± 4⋅19	77⋅65 ± 6⋅28	84⋅13 ± 5⋅81	83⋅74 ± 5⋅97	
	8	92⋅14 ± 5⋅11	84⋅83 ± 4⋅03	80⋅11 ± 6⋅26	79⋅95 ± 3⋅95	M: NS
	12	92⋅82 ± 5⋅54	84⋅08 ± 5⋅07	90⋅60 ± 5⋅94	87⋅16 ± 4⋅79	W: NS
	16	88⋅10 ± 3⋅20	87⋅73 ± 5⋅13	82⋅54 ± 5⋅35	79⋅82 ± 5⋅55	M × T: NS
Pulse, BPM	4	475⋅65 ± 17⋅63	453⋅66 ± 14⋅46	462⋅01 ± 8⋅68	473⋅74 ± 9⋅24	
	8	431⋅42 ± 9⋅43	463⋅22 ± 5⋅91	420⋅20 ± 6⋅91	418⋅71 ± 8⋅29	M: *P* = 0⋅07
	12	467⋅37 ± 10⋅21	445⋅08 ± 10⋅44	450⋅74 ± 13⋅33	457⋅83 ± 10⋅84	W: NS
	16	447⋅03 ± 11⋅03	434⋅67 ± 15⋅76	426⋅03 ± 12⋅06	416⋅71 ± 8⋅48	T: *P* < 0⋅0001

CD, casein diet; SD, soya protein diet; T, time; BPM, beats per minute, SBP, systolic blood pressure; DBP, diastolic blood pressure.

a MIXED model with maternal and weaning diet and time as main factors followed by Tukey's *post hoc* test when interaction was significant.

## Discussion

This study was the continuation of our previous studies investigating the effect of the source of protein in the maternal diet fed during gestation and lactation on the phenotype of the offspring of normal-weight pregnant rats. We previously reported that casein and soya protein as the sole source of protein in the maternal diet consumed during gestation and lactation by normal-weight Wistar rats influenced the health of mothers and their offspring differently. It was the first study showing that the source of protein in a nutritionally balanced diet may be a factor influencing the phenotype of the offspring. Maternal soya protein fed during gestation and lactation resulted in higher BW and systolic and diastolic blood pressure in offspring born to dams fed a soya protein diet compared to those born to dams fed a casein diet.^(36)^

Similarly, higher food intake was observed in offspring born to a dams-fed soya protein diet throughout post-weaning.^(38)^ Glucose homeostasis was also altered by the source of protein in the maternal diet: Higher fasting BG and glucose response to glucose load at week 12 and higher HOMA-IR at week 15 in offspring born to dams fed soya protein diet was observed.^(36)^

Although the results of the current study are consistent with our previous observation suggesting the role of the source of protein in the outcome of pregnancy, gestational obesity clearly had a dominant effect on the dietary proteins’ effect and masked their effects on various biomarkers to a certain extent. It can be explained by the fact that intrauterine exposure to maternal obesity is associated with an increased risk of metabolic syndrome and obesity in later life.^(39)^ More specifically, maternal obesity or GDM during pregnancy increases the risk of glucose intolerance in offspring.^(40)^

While the offspring's BW was influenced by the maternal dietary proteins in normal-weight mothers,^(38)^ no effect of maternal dietary proteins was observed in offspring born to obese mothers in this study. Moreover, contrasting our previous studies on normal-weight mothers,^(38)^ food intake was not influenced by the maternal diet but was influenced by the weaning diet. It was higher in offspring weaned to the soya protein diet. It could be due to the effect of weaning dietary proteins on offspring's food intake in a source-dependent manner. We previously reported that the source-dependent effect of proteins on food intake can be due to the individual characteristics of proteins including their digestion kinetics, amino acid composition, and sequence, and BAPs encrypted in their amino acid sequence.^(40)^ Although soya protein, as a fast protein, exhibits a stronger satiety effect in the short-term, casein, as a slow protein, has a more robust effect in the long term.^(41)^ The ineffectiveness of maternal dietary proteins on food intake can be due to the negative impact of gestational obesity on the food intake regulatory system through its *in utero* hyper-insulinemic effect on the development of the hypothalamus^(36)^ as a potential mechanism by which gestational obesity masked the effect maternal dietary proteins.

Glucose metabolism in offspring, unlike their BW and food intake, was influenced by maternal diet. Fasting BG, plasma concentrations of insulin, insulin/glucose ratio, and HOMA- IR were higher in pups born to SD dams. It is consistent with our previous observations in normal-weight mothers.^(36)^ The concentration of arginine, as one of the most potent insulinotropic amino acids,^(42–44)^ is almost twice in soya protein than casein and it may contribute to higher in utero insulin exposure of offspring of dams fed the S diet.^(36)^ Moreover, proline, which is twofold higher in casein compared with soya protein, may favourably alter glucose metabolism by stimulating glucose uptake in various tissues.^(45)^ It is well established that many BAPs in casein and soya protein affect the release of peptide hormones in the gastro-intestinal (GI) tract^(40)^ and are identified in blood.^(46)^ BAPs from both casein and soya protein exhibited anti-diabetic properties. Several BAPs extracted from soya glycinin showed favourable effects on glucose metabolism by increasing glucose uptake through activation of GLUT1 and GLUT4 by stimulation of AKT and AMPK pathways.^(47)^ Similarly, several BAPs from casein glycomacropeptide (GMP) enhanced glycogen synthesis via IRS-1/PI3K/Akt and AMPK signalling pathways. Moreover, tripeptides valine–proline–proline (VPP) and isoleucine–proline–proline (IPP), which are abundant in milk and dairy products, showed an insulin-sensitizing effect through enhancing Akt and ERK1/2 phosphorylation.^(47)^ However, as described earlier, the proline content of casein is twofold higher compared with soya protein, and it may have a direct effect on glucose metabolism and also an indirect effect via increasing the rate of absorption of BAPs rich in proline (e.g. VPP and IPP) encrypted in casein by increasing their resistance against digestion.

There was no effect of either gestational or weaning diet on blood pressure. It was clearly the overwriting of the effect of maternal dietary proteins by gestational obesity since in our previous study on normal-weight mothers, offspring born to the dams fed a soya protein-based diet showed higher SBP and DBP. It could be due to BAPs with a lowering effect on BP in casein. A daily dose of casein hydrolysate (0⋅49 g/d) which is abundant in BAPs peptides with lowering effect on BP (e.g. VPP and IPP), with inhibitory effects on angiotensin-converting enzyme, lowered both SBP and DBP.^(48)^ Oral administration of casein hydrolysate (32 mg/kg BW per d) reduced BP in hypertensive rats.^(49)^ However, gestational obesity elevated blood pressure in both dams’ groups mask the effect of the source of protein in the maternal diet. The effect of time on both SBP and pulse was significant indicating that both have been increased over time. Moreover, in the current study, an interactive effect of maternal and weaning diets on SBP was observed. Maternal dietary protein had an interactive but not synergistic effect on weaning dietary proteins. While SBP was relatively higher in pups weaned to the soya protein diet, it was also relatively higher in those who were born to dams fed a soya protein-based diet. Again, peptides known with a lowering effect on BP in casein in both maternal and weaning diets may explain, at least partially, this interaction.

The results of this study do not support the PAR hypothesis. Based on this hypothesis that offspring received a weaning diet that is matched with the maternal diet will have a more appropriate adaptation to the post-natal environment than those receiving an unmatched diet.^(38)^ Clearly, pups born to mothers fed a soya protein-based diet and weaned to the same diet did not show a better adaptation compared with those who received unmatched maternal and weaning diets. Although logistically it is extremely difficult, conducting a study including both normal-weight and obese mothers (comprising four groups of mothers and eight groups of pups) could provide a stronger comparability of obese *v.* normal-weight pregnant mothers and the pregnancy outcomes. It can be accounted as one of the limitations of this study since we had to compare the results of this study with our previous studies on normal-weight mothers conducted at different time and in different settings. Moreover, extending the duration of the study may help to examine the long-term consequences of gestational obesity in both mothers and offspring. It can be concluded based on the significant effect of the time in various measured parameters.

Although there is no direct implementation of these findings in humans, these results may be considered as a basis for future clinical trials since rodents and humans are sharing very similar metabolic and physiologic mechanisms.

## Conclusion

In conclusion, while gestational obesity either weakened or completely masked the effect of the source of protein in maternal diet on measured variables compared with our results from previous studies on normal-weight dams, the results of this study still support the notion that the source of protein in a nutritionally balanced diet may play a significant role in determining the outcome of the pregnancy even when counteracting with immense effects of gestational obesity on both mothers and their offspring.

## List of abbreviations

BW, body weight; BC, body composition; Cal, calorie; CD, casein diet; DBP, diastolic blood pressure; FBG, fasting blood glucose; FI, food intake; g, gram; IACUC, Institutional Animal Care and Use Committee; NS, not significant; OGTT, oral glucose tolerance test; SBP, systolic blood pressure; SD, soya protein diet; T, time.
